# Plantar Heel Pain Is Not Associated With Fatty Infiltration of the Abductor Digiti Minimi Muscle on Magnetic Resonance Imaging: A Cross‐Sectional Observational Study

**DOI:** 10.1002/jfa2.70155

**Published:** 2026-04-25

**Authors:** John S. C. Chen, John W. A. Osborne, Hylton B. Menz, Mandy Abbott, Shannon E. Munteanu, Tom Entwisle, David A. Connell, Karl B. Landorf

**Affiliations:** ^1^ Discipline of Podiatry, School of Allied Health, Human Services and Sport La Trobe University Melbourne Victoria Australia; ^2^ School of Health and Life Sciences Glasgow Caledonian University Glasgow Scotland UK; ^3^ Fowler Simmons Radiology Adelaide Australia; ^4^ Imaging@Olympic Park Melbourne Victoria Australia

**Keywords:** abductor digiti minimi, Baxter's neuropathy, fatty atrophy, fatty infiltration, magnetic resonance imaging, plantar fasciitis, plantar heel pain

## Abstract

**Background:**

Fatty infiltration, or fatty atrophy, of the abductor digiti minimi (ADM) muscle of the foot is proposed to be associated with entrapment of the first branch of the lateral plantar nerve (i.e., Baxter's neuropathy) as part of plantar heel pain (PHP). However, this association has not been rigorously investigated. The aim of this study was to determine if there is an association between PHP and fatty infiltration of ADM.

**Methods:**

This cross‐sectional observational study compared 50 participants with PHP to a control group of 25 participants without PHP who were matched at recruitment for age (± 5 years), sex and body mass index (BMI) (± 10%). Fatty infiltration of ADM was assessed on all participants using magnetic resonance imaging (MRI). Prior to assessment, four grading scales of fatty infiltration were investigated for reliability by two independent assessors using Kappa or weighted Kappa and the most reliable scale was chosen for the primary data analysis. Following this, participant characteristics were compared between the PHP and the no‐PHP groups using Chi‐squared, Mann‐Whitney *U* or independent samples *t*‐tests to ensure there were no significant differences between the two groups in characteristics that could have confounded the findings. The association between PHP and fatty infiltration was analysed using the Chi‐square test (*χ* ^2^).

**Results:**

In the PHP and no‐PHP groups, respectively, the mean age was 49.1 and 48.9 years, women comprised 58% and 56%, and the mean BMI was 30.6 and 30.2 kg/m^2^. A four‐point grading scale was found to be the most reliable scale (0.87 and 0.92 for inter‐ and intra‐rater reliability, respectively). There were no significant differences (*p* > 0.05) for important participant characteristics (e.g., age, sex and BMI). There was some fatty infiltration (grades 1–3) in 78% and 96% of participants in the PHP and no‐PHP groups respectively. No significant association was found (*χ* ^2^ = 6.176, df = 3, *p* = 0.103) between PHP and fatty infiltration according to the four‐point grading scale.

**Conclusions:**

After accounting for age, sex and BMI, there was no association between PHP and fatty infiltration of ADM. These findings suggest that adults with PHP do not have a greater predisposition for or amount of fatty infiltration of ADM compared to adults without PHP. Therefore, fatty infiltration of ADM is likely to be an incidental finding on MRI rather than a diagnostic sign of PHP, where Baxter's neuropathy may be present. Accordingly, clinicians should not focus on fatty infiltration of ADM on MRI to diagnose Baxter's neuropathy, or view it as a proxy marker, particularly when surgery for PHP is being considered.

**Abbreviations**
ADM: abductor digiti minimi
BMI: body mass index
CI: confidence interval
DICOM: digital imaging and communications in medicine
IBM: international business machines
IQR: interquartile range
MRI: magnetic resonance imaging
NiFTI: neuroimaging informatics technology initiative
PDW: proton density weighted
PHP: plantar heel pain
SD: standard deviation
SPAIR: spectral attenuated inversion recovery
STIR: short tau inversion recovery
STROBE: STrengthening the Reporting of OBservational studies in Epidemiology

## 1 Introduction

Plantar heel pain (PHP), also known as plantar fasciopathy or plantar fasciitis, refers to generalised pain at the plantar aspect of the heel [1]. It is one of the most prevalent foot disorders affecting up to 10% of the adult population [2, 3, 4], and is common in sedentary middle‐aged and older adults [2], long distance runners [5, 6] and military personnel [7]. PHP has been found to be associated with poorer generic and foot‐specific health‐related quality of life [8, 9], and increased levels of depression, anxiety and stress [10, 11, 12], all of which cause substantial economic burden [13].

**FIGURE 1:**
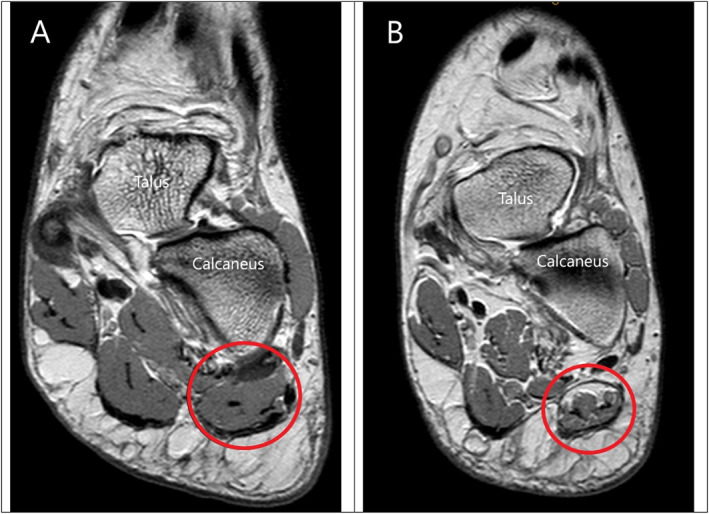
Examples of no fatty infiltration (image A) and fatty infiltration (image B) of the ADM muscle on coronal plane Proton Density Weighted (PDW) sequence MRIs. MRI scans are of the left foot (with the talus and calcaneus highlighted for orientation). The ADM muscle in both images lies inside the red circle. Within the ADM muscle, the medium signal intensity (grey) represents skeletal muscle and high signal intensity (white) represents fat [low signal intensity (black) represents fascia and tendon]. Image A illustrates an ADM with homogenous signal intensity representative of normal muscle. Image B illustrates an ADM with heterogenous signal intensity representative of fatty infiltration of the superior third of the muscle and mild fatty streaking in the inferior two thirds of the muscle.

Various causes of PHP have been described with plantar fasciopathy—pathology of the plantar fascia—cited as being the most common [14, 15, 16, 17]. Another commonly reported cause in up to 20% of cases of PHP is from a neural origin related to entrapment of the first branch of the lateral plantar nerve, which is referred to as ‘Baxter's neuropathy’ [18]. The first branch of the lateral plantar nerve is a mixed motor and sensory nerve that innervates abductor digiti minimi (ADM), flexor digitorum brevis and quadratus plantae, as well as providing sensory branches to the calcaneal periosteum and long plantar ligament [19, 20]. As it stands, there is no definitive diagnostic test for Baxter's neuropathy, such as a nerve conduction study, so fatty infiltration (or fatty atrophy) of the ADM muscle of the foot on magnetic resonance imaging (MRI)—Figure 1—has been hypothesised to be a proxy marker of the condition. The underlying hypothesis is that neuropathy of the first branch of the lateral plantar nerve—due to conditions associated with PHP such as plantar fasciopathy or plantar heel spur—leads to atrophy of ADM muscle tissue, which over time is replaced with fat. This hypothesis has been supported by several authors who have observed fatty infiltration of ADM in published case reports and case series studies [21, 22, 23, 24, 25, 26]. However, these study designs only provide low level evidence as they do not use a control group to minimise confounding, so their findings need to be viewed cautiously.

In addition to these case studies, there have been one retrospective and two cross‐sectional observational studies that have reported prevalence of fatty infiltration of ADM on MRI to be between 4% and 11% in the general population [27, 28, 29], with the prevalence being similar in adults with and without foot pain (approximately 8% and 6%, respectively) [29]. However, these studies included participants that had generalised foot pain, not PHP or Baxter's neuropathy specifically. Further, one of the cross‐sectional observational studies recruited an age‐ and sex‐matched asymptomatic control group, in addition to a foot pain group, but did not control for BMI [29], which could confound fatty infiltration of muscles as there is evidence to suggest that BMI [30], as well as age [31] and sex [32], influences fatty infiltration of skeletal muscle.

Importantly, from a clinical perspective, if the proxy marker of fatty infiltration of ADM is detected on MRI in an individual with PHP that has failed conservative treatment in the medium to long term (e.g., after 4–6 months), invasive surgery to release the first branch of the lateral plantar nerve has been recommended [33]. However, as outlined above, the evidence for this recommendation is poor. Accordingly, further rigorous investigation is needed to ascertain whether PHP is, indeed, associated with fatty infiltration of ADM on MRI. This would improve our understanding of the diagnostic value of MRI for this condition in clinical practice and may prevent unnecessary surgery for PHP if it is found not to be associated with fatty infiltration of ADM.

Therefore, this study aimed to investigate the association between PHP and fatty infiltration of ADM on MRI in adults with and without PHP, whilst controlling for age, sex and BMI.

## 2 Methods

Some of the methods outlined in this study have been reported in previous publications related to an over‐arching study that investigated associated factors relating to PHP [9, 16, 34].

### 2.1 Study Design

This study used a cross‐sectional observational design and was reported in accordance with the STrengthening the Reporting of OBservational studies in Epidemiology (STROBE) Statement [35].

### 2.2 Ethical Approval

Ethical approval was obtained for the over‐arching study by the La Trobe University (LTU) Human Research and Ethics Committee, Melbourne, Australia (Application 14‐001) on the 21st of August 2014. Ethical approval was also retrospectively obtained from the Glasgow Caledonian University Research Ethical Committee, Glasgow, Scotland (Application HLS/PSWAHS/23/226) on the 9th of April 2024—this was a requirement for the data to be used in the first author's (JSCC) Masters of Science dissertation completed through this university.

### 2.3 Participants

The participants were 75 community‐dwelling adults of either sex from the State of Victoria, Australia. There were two groups of participants: (i) a case group of 50 participants with PHP, and (ii) a control group of 25 matched participants without PHP (i.e., a ratio of 2:1 cases to controls). Participants in the control group were matched to the case group by age (± 5 years), sex and BMI (± 10%) [9, 16, 34].

### 2.4 Eligibility Criteria

Participants were eligible if they:

- Were aged 18 years or over;
- Had PHP for at least 1 month (if recruited to the PHP group);
- Could speak basic English, so they could provide informed consent prior to participation, follow instructions during the project and answer questions related to the study accurately.

Participants were ineligible if they had:

- Any conditions (e.g., pregnancy, pacemaker, metal fragments etc.) that precluded them from having the medical imaging;
- Any self‐reported inflammatory arthritis (e.g., seronegative arthropathy), endocrine or neurological condition (e.g., diabetic peripheral neuropathy) or surgery (e.g., joint fusion) that affected lower limb sensation or their ability to walk or run.

### 2.5 Recruitment

Participants were recruited via several methods including: advertising posters placed at relevant locations (e.g., LTU, private and public health clinics, sporting and senior citizen clubs), the Health Sciences Clinic at LTU, advertisements on relevant websites related to health, direct referral from health care practitioners, and via acquaintances of the investigators involved with the study and snowball sampling. Recruitment commenced on 12th of January 2015 and was completed on 26th of October 2018.

### 2.6 Data Collection

Participant characteristic data including age, sex, BMI, general medical history and physical characteristics were collected using a standardised assessment form (Supporting Information S1) by one of three registered podiatrists, all of whom had more than 10 years of experience at the time of data collection. Following written informed consent, participants were examined in one session that took approximately one to one and a half hours, and then within 1 week, they attended a medical imaging clinic (Imaging @ Olympic Park, located at AAMI Park, Melbourne, Australia) to have MRI scans of both feet (focused on their ankles, heels and midfoot).

### 2.7 MRI Protocol

MRI scans were generated using a Philips 3.0 Achieva Tesla scanner. Scans of both feet were taken in an 8‐channel foot/ankle receive coil in two planes, sagittal and coronal of the rearfoot only, excluding the metatarsals and phalanges. Sagittal plane images included Proton Density Weighted (PDW) (800 × 800) and PDW—SPectral Attenuated Inversion Recovery (SPAIR) sequences (512 × 512). Coronal plane images included PDW (800 × 800) and Short Tau Inversion Recovery (STIR) sequences (432 × 432). Slice thicknesses were: 1.5 mm for the PDW sequence and 2.0 mm for SPAIR sequence in the sagittal plane, and 1.5 mm for PDW sequence and 2.5 mm for STIR sequence in the coronal plane. This study focused on the coronal plane PDW and STIR sequences to assess the frequency and extent of fatty infiltration of ADM on MRI.

### 2.8 MRI Processing and Assessment

MRI data were exported in Digital Imaging and Communications in Medicine (DICOM) format, anonymised via DICOManonymizer (i.e., participants identifying details were removed from each MRI scan), converted to Neuroimaging Informatics Technology Initiative (NiFTI) format and exported to 3D imaging software (ITK‐SNAP) for image assessment.

To assess each participant's MRIs, the raters were, after opening the appropriate MRI sequence, required to choose the MRI slice that displayed the maximum amount of fatty infiltration of ADM (if present). The raters focused on reviewing coronal plane PDW images to assess the presence of fatty infiltration of ADM, but they could also use the other available MRI sequences and plane to assist with their assessment.

A fatty infiltration grading tool was developed for all gradings of fatty infiltration from the MRIs (Supporting Information S2). Initially, a simple dichotomous scale (i.e., a ‘yes’ or ‘no’) was used to assess the presence of fatty infiltration of ADM on MRI. Subsequently, three ordinal grading scales [28, 29, 36] were used to categorise the extent of fatty infiltration of ADM on MRI—Table 1.

**TABLE 1:** Grading scales to assess fatty infiltration of ADM on MRI in adults with and without PHP.

Classification	Grade 0	Grade 1	Grade 2	Grade 3	Grade 4
Schmid et al. (2009)	Normal muscle	Mild fatty atrophy with more muscle than fat	Substantial fatty atrophy with more fat than muscle or equal parts of fat and muscle	No grade 3 in this scale	No grade 4 in this scale
Recht et al. (2007)	No fat or minimal fatty streaks	Increased fat within the muscle but greater amount of muscle	Equal amounts of fat and muscle	Greater amount of fat than muscle	No grade 4 in this scale
Goutallier et al. (1994)	Completely normal without any fatty streaks	Muscle contains some fatty streaks	Fatty infiltration is important, but there is more muscle than fat	Equal amounts of fat and muscle	More fat than muscle is present

### 2.9 Reliability Testing

Prior to the final assessment of the MRIs, 20 participants were randomly chosen from the PHP group for reliability testing. In addition to the MRI files being anonymised and to enable blinded assessment, the order of participants was randomly shuffled by one of the authors who was not one of the raters (KBL). Reliability testing was performed by two raters (two of the authors, JSCC and JWAO), both registered podiatrists with 6 and 11 years of experience at the time of the study, respectively. Both raters had substantial training in MRI interpretation from the chief investigator of the over‐arching study (KBL) who had more than 10 years of research experience using MRI at the time of the study. The MRIs were initially assessed by the two raters for inter‐rater agreement, and then the same 20 participants were re‐assessed one week later by one rater (JSCC) for intra‐rater agreement. Once satisfactory reliability of the scales was determined, the primary rater (JSCC) applied the scale with the highest reliability to assess all remaining 55 MRI scans in the overall dataset of 75 participants.

### 2.10 Data Analysis

Data analysis was undertaken using International Business Machines (IBM) Statistical Package for the Social Sciences version 29.0 (IBM Corp, Somers, NY, USA). Only measurements of the left foot were analysed, so that the assumption of independence for statistical analysis was adhered to [37, 38]. Reliability of the scales was assessed using Kappa (for dichotomous scales) and weighted Kappa (for ordinal scales) statistical tests. Quadratic weights were used in the estimation of weighted Kappas. Participant characteristics were compared between the PHP and the no‐PHP groups using Chi‐squared, Mann‐Whitney *U* or independent samples *t*‐tests depending on the scale of the data. Continuous participant characteristic data were checked for normal distribution prior to parametric statistical testing. Chi‐square tests were used to test whether there was an association between PHP and fatty infiltration of the ADM on MRI. The likelihood ratio was used for the Chi‐square test where greater than 20% of cells had an expected count of less than 5. The critical level of significance was set at *p* < 0.05 and 95% confidence intervals (95% CIs) were calculated where appropriate.

## 3 Results

### 3.1 Participant Characteristics

Seventy‐five participants were recruited into the study. There were 50 participants with PHP and 25 participants without PHP that were matched for age, sex and BMI. There were no statistically significant differences for age, sex or BMI between the two groups (Table 2), so the matching methods used at recruitment were successful. The mean age was 49.1 years in the PHP group and 48.9 years in the no‐PHP group, with a range for all participants of 23–75 years. Women comprised 58% of the PHP group and 56% of the no‐PHP group. The mean BMI was 30.6 kg/m^2^ in the PHP group and 30.2 kg/m^2^ in the no‐PHP group. Therefore, participants were on average, obese, although the BMI ranged from normal to very severely obese (range 20.1–47.7 kg/m^2^). In addition to BMI, the measure of abdominal obesity (via the waist‐hip ratio) was also well matched between the two groups, as were education level, number of self‐reported medications, activity levels, and medical conditions that may have been associated with fat deposition.

**TABLE 2:** Participant characteristics—Values are means (SDs) unless otherwise stated.

Variable	PHP group (*n* = 50)	No‐PHP group (*n* = 25)	Mean difference (95% CI)	*p*‐value
Age (years)	49.1 (11.6)	48.9 (9.9)	0.2 (−5.2 to 5.6)	0.947
Sex—no. of women (%)	29 (58%)	14 (56%)	N/A	0.869^a^
Height (m)	1.68 (0.10)	1.73 (0.12)	−0.05 (−0.11 to 0.00)	0.051
Weight (kg)	86.1 (17.5)	90.3 (21.4)	−4.2 (−13.4 to 5.0)	0.370
BMI (kg/m^2^)	30.6 (6.2)	30.2 (7.2)	0.4 (−2.8 to 3.6)	0.813
Waist circumference (cm)	100.9 (11.4)	101.7 (19.4)	−0.8 (−9.3 to 7.8)^b^	0.858^b^
Hip circumference (cm)	112.0 (12.6)	111.1 (15.3)	0.9 (−5.7 to 7.5)	0.784
Waist‐hip ratio	0.90 (0.06)	0.91 (0.09)	−0.01 (−0.05 to 0.03)	0.614
Duration of symptoms				
Median (IQR)^c^	6.5 (3.0–12.0)	0.0 (0.0–0.0)	N/A	N/A
Education level—Category				
Median (IQR)^c^	6 (4–7)	6 (5–6.5)	N/A	0.785^d^
No. of prescribed medications				
Median (IQR)^e^	0 (0–1)	0 (0–1)	N/A	0.430^d^
Activity level (kilocalories expended per day)^f^	3745 (1012)	3689 (1034)	56 (−441 to 554)	0.823
Medical conditions				
Diabetes^g^	1 (2%)	1 (4%)	N/A	0.612^a^
Hypertension	5 (10%)	2 (8%)	N/A	0.779^a^
Heart disease	1 (2%)	1 (4%)	N/A	0.612^a^
Hormone replacement therapy	1 (2%)	1 (4%)	N/A	0.612^a^
Hypercholesterolaemia	2 (4%)	3 (12%)	N/A	0.190^a^
Peripheral vascular disease	0 (0%)	0 (0%)	N/A	N/A

Abbreviation: N/A = Not applicable.

^a^ *p*‐value relates to Chi‐squared test.

^b^ Mean difference, 95% CIs and *p*‐value adjusted as Levene's Test for Equality of Variances was significant (*p* < 0.05).

^c^ Education level represents the highest level of education that participants had completed, which was categorised as: (i) no formal, (ii) less than primary school, (iii) primary school completed, (iv) high school (or equivalent) completed, (v) TAFE (technical college) completed, (vi) college/university completed, (vii) post graduate degree completed, (viii) don't know, (ix) other (please state).

^d^ *p*‐value relates to Mann‐Whitney *U* test.

^e^ Median (IQR) reported as variable not normally distributed.

^f^ Activity level was measured by the Stanford Activity Questionnaire, which was expressed as kilocalories expended per day [39, 40].

^g^ Cases did not have any related condition (e.g., peripheral neuropathy) affecting lower limb sensation or their ability to walk/run.

In the PHP group, the median duration of symptoms ranged between 1.0 and 80.0 months, with a median duration of 6.5 months. Mean first step pain on a 100 mm VAS was 53 mm, mean pain on the day of their assessment was 39 mm, and mean pain in the last 7 days was 50 mm.

### 3.2 Inter‐and Intra‐Rater Reliability

The inter‐ and intra‐rater reliability of measurements for the four fatty infiltration grading scales is shown in Table 3. The best reliability was found for the grading scale proposed by Recht et al. [28]. Therefore, this grading scale was chosen as being the most reliable to assess all 75 participants.

**TABLE 3:** Inter‐ and intra‐rater reliability results (with 95% CIs) for the four grading scales initially assessed (*n* = 20).

Grading scale	Inter‐rater reliability	Intra‐rater reliability
Dichotomous (yes/no)^a^	0.22 (−0.33 to 0.76)	0.46 (−0.14 to 1.00)^b^
Schmid et al. (2009)^c^	0.71 (0.45 to 0.97)	0.83 (0.61 to 1.00)^b^
Recht et al. (2007)^c^	0.87 (0.74 to 1.00)^b^	0.92 (0.83 to 1.00)^b^
Goutallier et al. (1994)^c^	0.85 (0.69 to 1.00)^b^	0.89 (0.78 to 0.99)

^a^ The Kappa statistic was used to assess reliability as grading scale had only two categories.

^b^ Upper confidence interval has been truncated to 1.00 (i.e., 100%).

^c^ Weighted Kappa used to assess agreement as grading scale had more than two categories.

### 3.3 Frequency of Fatty Infiltration of ADM in Adults With PHP

Table 4 presents the cross‐tabulations for each of the Recht et al. grades for the PHP and no‐PHP groups. There was no significant association between PHP and fatty infiltration of ADM (*χ* ^2^ = 6.176, df = 3, *p* = 0.103).

**TABLE 4:** Crosstabulations for Recht et al. grades for the PHP and no‐PHP groups.

Recht et al. grade	PHP group *n* (%)	No‐PHP group *n* (%)
Grade 0—No fat or minimal fatty streaks	11 (22%)	1 (4%)
Grade 1—Increased fat but greater amount of muscle	34 (68%)	20 (80%)
Grade 2—Equal amounts of fat and muscle	2 (4%)	3 (12%)
Grade 3—Greater amount of fat than muscle	3 (6%)	1 (4%)

*Note:* greater than 20% of cells had an expected count of less than 5, so the likelihood ratio was used for the *χ* ^2^ test.

**FIGURE 2:**
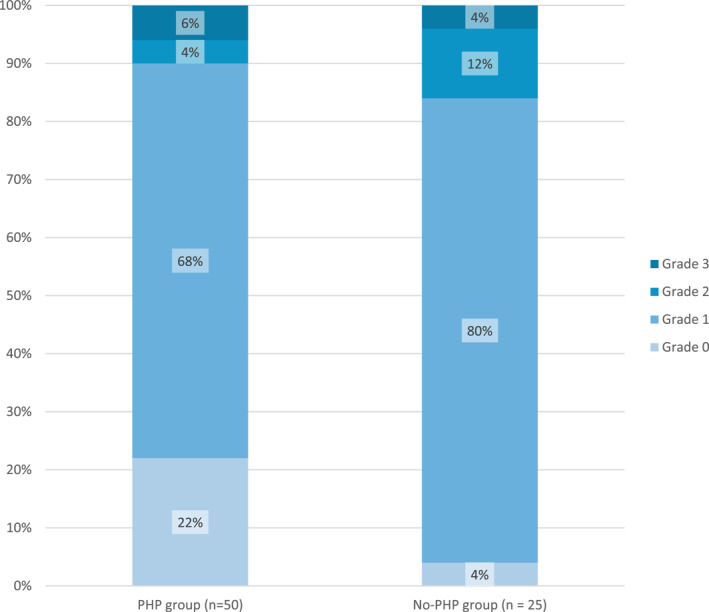
Stacked bar chart of the percentage of participants in the two groups for each grade of the Recht et al. scale.

Figure 2 presents the data in Table 4 in a stacked bar chart, so the percentages in each grade for the two groups can be compared graphically.

## 4 Discussion

This study aimed to investigate the association between PHP and fatty infiltration of ADM on MRI, which we believe is the first study to do so. To achieve our aim, a group of participants with PHP were compared to a group of participants without PHP who were matched for age, sex and BMI. BMI is important in the context of this study because it is associated with fatty infiltration of skeletal muscle [30]. In addition to BMI, the groups had similar abdominal obesity (via the waist‐hip ratio), which is another important measure of obesity, and frequency of medical conditions associated with obesity (e.g., diabetes and hypercholesterolaemia). Hence, potential confounding of fatty infiltration in ADM due to increased BMI and abdominal adiposity was minimised. After determining the participants were well matched, the association of PHP and fatty infiltration of ADM was investigated using a four‐point grading scale developed by Recht et al. [28], which demonstrated the best reliability between scales that were initially considered [28, 29, 36].

This study found that there was not a significant association between PHP and fatty infiltration of ADM. Accordingly, our findings suggest that adults with PHP do not have a greater predisposition for or amount of fatty infiltration of ADM compared to adults without PHP. Because there was no significant association between PHP and fatty infiltration (i.e., no difference between the groups in the grades of fatty infiltration), additional dichotomisation of the grades into ‘no fat’ versus ‘fat’ within the muscle was not carried out. This was further supported by the finding that more participants in the PHP group had a finding of Grade 0 (i.e., no fat) compared with the no‐PHP group, which was opposite to that expected if the fatty infiltration hypothesis were true. In addition, due to the non‐significant finding (i.e., there was no significant association between PHP and fatty infiltration), odds ratios and 95% CIs were not calculated.

Our finding contrasts with what has been suggested by other investigators. Stanzcak and colleagues [41] were the first to report in 2001 fatty infiltration of ADM on MRI. They compared 10 participants with fatty infiltration of ADM to a control group of 19 age‐ and sex‐matched participants who did not have fatty infiltration of ADM. They found that advanced fatty infiltration of ADM was more likely if individuals had heel pain, heel spurs and/or paraesthesia in the region of the heel. Therefore, they concluded that "advanced ADM muscle atrophy supports the existence of a true Baxter's neuropathy" (p 522). However, the control group had a significantly lower BMI, so the findings may have been confounded by obesity. Moreover, their findings were presented as a published conference abstract only and as far as we can ascertain, they have not progressed to a full peer‐reviewed journal article. Consequently, there is limited methodological information available, so we cannot confidently compare our findings to theirs any further.

Since then, Dirim and colleagues published their observations of fatty infiltration of ADM in an individual with bilateral PHP [21]. They attributed their findings in this single case study to Baxter's neuropathy secondary to plantar fasciopathy, writing "selective fatty atrophy of the abductor digiti minimi muscle is the unique sign of this neuropathy" (p CS53) [21]. From their observations, they suggested that entrapment of the first branch of the lateral plantar nerve can lead to fatty infiltration of the ADM muscle being visible on MRI [21], which was hypothesised to be a proxy marker of nerve entrapment of the first branch of the lateral plantar nerve. This hypothesis has since been supported by other case studies [22, 23, 24, 25, 26]. Our contrasting finding of no association between PHP and fatty infiltration of ADM can be explained by the methodology used in these case studies, which provide no controls, so they represent lower‐level evidence than our study.

Other than the studies discussed above, three additional studies (one retrospective and two cross‐sectional observational studies) have reported the prevalence of fatty infiltration of ADM on MRI in adults with foot pain from a variety of conditions [27, 28, 29]. Although of interest, these studies did not focus on PHP in isolation and did not investigate the association between PHP and fatty infiltration of ADM. Nevertheless, they provide interesting insights into fatty infiltration of the ADM. The first of these, a cross‐sectional observational study by Recht and colleagues [28], recruited a large sample of 602 consecutive participants with foot pain from a variety of conditions (not just PHP) and reported a 6.3% (38/602) prevalence of fatty infiltration of ADM on MRI (grades 1–3 on the Recht et al. grading scale). Somewhat surprisingly, the authors found that none of the participants with fatty infiltration of ADM presented with a possible diagnosis of Baxter's neuropathy (i.e., participants with fatty infiltration of ADM had no clinical signs and symptoms of entrapment of the first branch of the lateral plantar nerve). As a result, they concluded that fatty infiltration of ADM may be an incidental finding on MRI and unrelated to nerve entrapment.

The second cross‐sectional observational study by Schmid and colleagues [29] recruited 160 participants, including 80 with foot pain from a variety of conditions and 80 without foot pain who were matched for age and sex. They observed a similar prevalence of substantial fatty infiltration of ADM on MRI between those with foot pain (approximately 8%) and those without foot pain (approximately 6%), which was found to be not significantly different [29]. However, Schmid and colleagues [29] did not account for increased BMI or abdominal obesity, so these factors cannot be ruled out as confounding their findings. Furthermore, this study [29] did not check to ensure the groups were similar for other important co‐morbidities that could have confounded their findings (e.g., medical conditions that may be associated with fat deposition).

The third study by Chundru and colleagues [27] was retrospective, not cross‐sectional like the two studies discussed above or our study. They also investigated the prevalence of fatty infiltration of ADM from 1780 MRI reports from community‐dwelling adults with generalised foot pain. They found a similar prevalence in their sample to Recht et al. [28] of 5.6% for fatty infiltration of ADM. Following their prevalence calculation, they went on to further analyse 100 individuals with and without fatty infiltration of ADM and found a significant association between fatty infiltration of ADM and advancing age and two conditions associated with PHP, plantar calcaneal spur formation and plantar fasciopathy [27]. However, their findings may have been confounded by BMI as they did not account for that, as we did.

With the previous studies outlined above in mind, our finding of no association between PHP and fatty infiltration of ADM is difficult to compare for several reasons. Firstly, the case studies did not have a control group and as such, are prone to confounding and bias. Secondly, and as highlighted above, the retrospective and cross‐sectional studies recruited participants with foot pain regardless of the underlying cause [27, 28, 29]. It is therefore most likely that their observations relating to fatty infiltration of ADM are not attributable to PHP and Baxter's neuropathy. Thirdly, previous studies that did grade fatty infiltration of ADM used different grading scales compared with the four‐point Recht et al. [28] grading scale that we used in our study. As a result, direct comparisons cannot be made between studies as interpretations and gradings of fatty infiltration of ADM are not consistent. Lastly, most previous studies did not undertake reliability testing for raters prior to measurement [27, 28], so their reliability is unknown. Schmid and colleagues [29] did attempt to overcome this by conducting "interobserver agreement" for the grading of fatty muscle atrophy (fatty infiltration) between raters but only did this using a three‐point grading scale that they developed. By contrast, our study has conducted reliability testing on all previously published ordinal scales that were initially considered and then chose the most reliable grading scale [28].

There are three strengths to our study. Firstly, our study matched a general sample of adult participants with and without PHP for age, sex and BMI. This is important because BMI, which most other studies did not account for, has been consistently associated with PHP [42, 43], and to a lesser extent, fatty infiltration of skeletal muscle [30], so it may be a confounding factor. Secondly, this study performed inter‐ and intra‐rater reliability testing for each of the grading scales and found that raters had best reliability using the Recht et al. [28] grading scale. This approach ensured reliable assessment methods were used for grading fatty infiltration of ADM on MRI to improve the internal validity of findings. Lastly, the MR images that were analysed in this study were taken with a 3.0 T MRI scanner, which has a higher field‐strength, leading to better image quality for assessment of anatomical structures including muscle [44].

This study also has three limitations that need to be considered. Firstly, the sample size for this study was relatively small (*n* = 75) as it was dictated by the over‐arching study, which was limited in its recruitment by the expensive imaging modalities that were used on all participants [9, 16, 34]. As a result, some of the cell counts in our analysis were small (i.e., the numbers of participants with certain grades of fatty infiltration was low), which could have affected some approximations in our analysis. However, we used the likelihood ratio where appropriate, which provides better approximations than Chi‐square with smaller samples. Further studies with larger sample sizes would be beneficial to add greater statistical precision. Secondly, participants in this study were recruited from a wider population of community‐dwelling adults with PHP, so the results are generalisable to this population and not specific sub‐populations with PHP (e.g., younger adults, long distance runners or military recruits who have been found to have relatively high prevalence of PHP). Finally, this study recruited adults with a clinical diagnosis of PHP without a specific diagnosis of Baxter's neuropathy (i.e., entrapment of the first branch of the lateral plantar nerve), which is currently not feasible using non‐invasive testing and is, therefore, not performed in clinical practice. Although further research would be ideal, such investigation for specific nerve entrapment of this nerve is complex and is not routinely available or definitive. Accordingly, we cannot conclude for certain that adults with a specific diagnosis of Baxter's neuropathy via laboratory‐based nerve conduction studies would not find a different conclusion.

## 5 Conclusions

This study aimed to determine if PHP is associated with fatty infiltration of ADM on MRI in adults. The findings revealed no significant association between PHP and fatty infiltration of ADM after accounting for age, sex and BMI. Therefore, fatty infiltration of ADM may be an incidental finding on MRI rather than a diagnostic sign of Baxter's neuropathy in adults with PHP. Accordingly, this study recommends that clinicians should not rely on fatty infiltration of ADM on MRI to inform their clinical diagnosis of Baxter's neuropathy, especially when surgery, such as nerve decompression, is being considered for recalcitrant PHP.

## Author Contributions

**John S. C. Chen:** conceptualization, methodology, investigation, data curation, formal analysis, validation, visualization, writing – original draft preparation, writing – review and editing. **John W. A. Osborne:** investigation, methodology, validation, writing – review and editing. **Hylton B. Menz:** conceptualization, supervision, methodology, data curation, validation, formal analysis, visualization, writing – review and editing. **Mandy Abbott:** supervision, writing – review and editing. **Shannon E. Munteanu:** conceptualization, methodology, writing – review and editing. **Tom Entwisle:** conceptualization, methodology, writing – review and editing. **David A. Connell:** conceptualization, methodology, writing – review and editing. **Karl B. Landorf:** conceptualization, project administration, supervision, methodology, investigation, data curation, formal analysis, validation, visualization, writing – original draft preparation, writing – review and editing.

## Funding

This project was funded from research grants provided by the Sport, Exercise and Rehabilitation Research Focus Area at La Trobe University and the School of Allied Health at La Trobe University.

## Ethics Statement

Ethical approval was obtained from the La Trobe University Human Ethics Committee—Application 14‐001. Ethical approval was also obtained from the Glasgow Caledonian University Research Ethical Committee, Glasgow, Scotland—Application HLS/PSWAHS/23/226.

## Consent

All participants provided written informed consent prior to recruitment into the study.

## Conflicts of Interest

The authors declare no conflicts of interest.

## Supporting information


Supporting Information S1

Supporting Information S2

## Data Availability

The datasets generated during and/or analysed during the current study are available from the corresponding and senior/last authors on reasonable request.

## References

[1] R. Buchbinder , “Clinical Practice Plantar Fasciitis,” New England Journal of Medicine 350, no. 21 (2004): 2159–2166, 10.1056/NEJMcp032745. 15152061

[2] J. E. Dunn , C. L. Link , D. T. Felson , M. G. Crincoli , J. J. Keysor , and J. B. McKinlay , “Prevalence of Foot and Ankle Conditions in a Multiethnic Community Sample of Older Adults,” American Journal of Epidemiology 159, no. 5 (2004): 491–498, 10.1093/aje/kwh071. 14977645

[3] C. L. Hill , T. K. Gill , H. B. Menz , and A. W. Taylor , “Prevalence and Correlates of Foot Pain in a Population‐Based Study: The North West Adelaide Health Study,” Journal of Foot and Ankle Research 1, (2008): 2, 10.1186/1757-1146-1-2. 18822153 PMC2547889

[4] M. J. Thomas , E. Roddy , W. Zhang , H. B. Menz , M. T. Hannan , and G. M. Peat , “The Population Prevalence of Foot and Ankle Pain in Middle and Old Age: A Systematic Review,” Pain 152, no. 12 (2011): 2870–2880, 10.1016/j.pain.2011.09.019. 22019150

[5] W. B. Kibler , C. Goldberg , and T. J. Chandler , “Functional Biomechanical Deficits in Running Athletes With Plantar Fasciitis,” American Journal of Sports Medicine 19, no. 1 (1991): 66–71, 10.1177/036354659101900111. 1672577

[6] B. L. Warren , “Anatomical Factors Associated With Predicting Plantar Fasciitis in Long‐Distance Runners,” Medicine & Science in Sports & Exercise 16, no. 1 (1984): 60–63, 10.1249/00005768-198401000-00012. 6708780

[7] M. Sadat‐Ali , “Plantar Fasciitis/Calcaneal Spur Among Security Forces Personnel,” Military Medicine 163, no. 1 (1998): 56–57, 10.1093/milmed/163.1.56. 9465574

[8] D. B. Irving , J. L. Cook , M. A. Young , and H. B. Menz , “Impact of Chronic Plantar Heel Pain on Health‐Related Quality of Life,” Journal of the American Podiatric Medical Association 98, no. 4 (2008): 283–289, 10.7547/0980283. 18685048

[9] K. B. Landorf , M. R. Kaminski , S. E. Munteanu , G. V. Zammit , and H. B. Menz , “Health‐Related Quality of Life Is Substantially Worse in Individuals With Plantar Heel Pain,” Scientific Reports 12, no. 1 (2022): 15652, 10.1038/s41598-022-19588-5. 36123358 PMC9485111

[10] M. Cotchett , S. E. Munteanu , and K. B. Landorf , “Depression, Anxiety, and Stress in People With and Without Plantar Heel Pain,” Foot & Ankle International 37, no. 8 (2016): 816–821, 10.1177/1071100716646630. 27137796

[11] M. Cotchett , A. Lennecke , V. G. Medica , G. A. Whittaker , and D. R. Bonanno , “The Association Between Pain Catastrophising and Kinesiophobia With Pain and Function in People With Plantar Heel Pain,” Foot 32 (2017): 8–14, 10.1016/j.foot.2017.03.003. 28605621

[12] C. Drake , A. Mallows , and C. Littlewood , “Psychosocial Variables and Presence, Severity and Prognosis of Plantar Heel Pain: A Systematic Review of cross‐sectional and Prognostic Associations,” Musculoskeletal Care 16, no. 3 (2018): 329–338, 10.1002/msc.1246. 29766646

[13] K. B. Tong and J. Furia , “Economic Burden of Plantar Fasciitis Treatment in the United States,” American Journal of Orthopedics 39, no. 5 (2010): 227–231. 20567740

[14] D. B. Irving , J. L. Cook , and H. B. Menz , “Factors Associated With Chronic Plantar Heel Pain: A Systematic Review,” Journal of Science and Medicine in Sport 9, no. 1–2 (2006): 11–22, 10.1016/j.jsams.2006.02.004. 16584917

[15] D. B. Irving , J. L. Cook , M. A. Young , and H. B. Menz , “Obesity and Pronated Foot Type May Increase the Risk of Chronic Plantar Heel Pain: A Matched Case‐Control Study,” BMC Musculoskeletal Disorders 8, no. 1 (2007): 41, 10.1186/1471-2474-8-41. 17506905 PMC1884155

[16] K. B. Landorf , M. R. Kaminski , S. E. Munteanu , G. V. Zammit , and H. B. Menz , “Activity and Footwear Characteristics in People With and Without Plantar Heel Pain: A Matched Cross‐Sectional Observational Study,” Musculoskeletal Care 21, no. 1 (2023): 35–44, 10.1002/msc.1663. 35678543

[17] D. L. Riddle , M. Pulisic , P. Pidcoe , and R. E. Johnson , “Risk Factors for Plantar Fasciitis: A Matched Case‐Control Study,” Journal of Bone and Joint Surgery. American 85, no. 5 (2003): 872–877, 10.2106/00004623-200305000-00015. 12728038

[18] D. E. Baxter , G. B. Pfeffer , and M. Thigpen , “Chronic Heel Pain. Treatment Rationale,” Orthopedic Clinics of North America 20, no. 4 (1989): 563–569. 2797751

[19] M. del Sol , E. Olave , C. Gabrielli , E. Mandiola , and J. C. Prates , “Innervation of the Abductor Digiti Minimi Muscle of the Human Foot: Anatomical Basis of the Entrapment of the Abductor Digiti Minimi Nerve,” Surgical and Radiologic Anatomy 24, no. 1 (2002): 18–22, 10.1007/s00276-002-0001-1. 12197005

[20] J. J. Rondhuis and A. Huson , “The First Branch of the Lateral Plantar Nerve and Heel Pain,” Acta Morphologica Neerlando‐Scandinavica 24, no. 4 (1986): 269–279. 3425404

[21] B. Dirim , D. Resnick , and N. K. Ozenler , “Bilateral Baxter’s Neuropathy Secondary to Plantar Fasciitis,” Medical Science Monitor 16, no. 4 (2010): CS50–CS53. 20357723

[22] M. Chimutengwende‐Gordon , P. O'Donnell , N. Cullen , and D. Singh , “Oedema of the Abductor Digiti Quinti Muscle Due To Whom It May Concern: Subacute Denervation: Report of Two Cases,” Foot and Ankle Surgery 20, no. 1 (2014): e3–e6, 10.1016/j.fas.2013.09.002. 24480510

[23] M. R. F. Jaring , A. Z. Khan , J. A. Livingstone , and J. Chakraverty , “A Case of Bilateral Baxter's Neuropathy Secondary to Plantar Fasciitis,” Journal of Foot and Ankle Surgery 58, no. 4 (2019): 771–774, 10.1053/j.jfas.2018.11.010. 31027970

[24] H. Kaur , P. Tiwari , and N. Bansal , “Plantar Fasciitis With Chronic Baxter's Neuropathy Causing Hindfoot Pain—A Case Report,” Journal of Orthopaedic Case Reports 14, no. 2 (2024): 150–154, 10.13107/jocr.2024.v14.i02.4252. PMC10898688 38420250

[25] M. S. Moreno García , P. S. del Río‐Martínez , N. Yanguas Barea , and P. Baltanás Rubio , “Hindfoot Pain: Baxter Neuropathy,” Reumatología Clínica 13, no. 2 (2017): 123, 10.1016/j.reumae.2016.05.003. 27365275

[26] C. Y. G. Ong and T. Y. Chin , “Clinics in Diagnostic Imaging (205). Baxter's Neuropathy,” Singapore Medical Journal 61, no. 4 (2020): 176–180, 10.11622/smedj.2020055. 32500156 PMC7905142

[27] U. Chundru , A. Liebeskind , F. Seidelmann , J. Fogel , P. Franklin , and J. Beltran , “Plantar Fasciitis and Calcaneal Spur Formation Are Associated With Abductor Digiti Minimi Atrophy on MRI of the Foot,” Skeletal Radiology 37, no. 6 (2008): 505–510, 10.1007/s00256-008-0455-2. 18286281 PMC2335296

[28] M. P. Recht , P. Grooff , H. Ilaslan , H. S. Recht , J. Sferra , and B. G. Donley , “Selective Atrophy of the Abductor Digiti Quinti: An MRI Study,” AJR. American Journal of Roentgenology 189, no. 3 (2007): W123–W127, 10.2214/AJR.07.2229. 17715077

[29] D. T. Schmid , J. Hodler , B. Mengiardi , C. W. Pfirrmann , N. Espinosa , and M. Zanetti , “Fatty Muscle Atrophy: Prevalence in the Hindfoot Muscles on MR Images of Asymptomatic Volunteers and Patients With Foot Pain,” Radiology 253, no. 1 (2009): 160–166, 10.1148/radiol.2531090035. 19703848

[30] J. A. Pasco , S. X. Sui , E. C. West , et al., “Fatty Liver Index and Skeletal Muscle Density,” Calcified Tissue International 110, no. 6 (2022): 649–657, 10.1007/s00223-021-00939-9. 35028685 PMC9108103

[31] R. J. Crawford , L. Filli , J. M. Elliott , et al., “Age‐ and Level‐Dependence of Fatty Infiltration in Lumbar Paravertebral Muscles of Healthy Volunteers,” AJNR. American Journal of Neuroradiology 37, no. 4 (2016): 742–748, 10.3174/ajnr.A4596. 26635285 PMC7960169

[32] M. V. Douma , N. C. Casartelli , R. Sutter , M. Leunig , and N. A. Maffiuletti , “Sex‐Specific Differences in Hip Muscle Cross‐Sectional Area and Fatty Infiltration in Patients With Femoroacetabular Impingement Syndrome,” Orthopaedic Journal of Sports Medicine 11, no. 1 (2023): 23259671221147528, 10.1177/23259671221147528. 36743730 PMC9893369

[33] D. E. Baxter and G. B. Pfeffer , “Treatment of Chronic Heel Pain by Surgical Release of the First Branch of the Lateral Plantar Nerve,” Clinical Orthopaedics and Related Research 279 (1992): 229–236, 10.1097/00003086-199206000-00029. 1600660

[34] K. B. Landorf , M. R. Kaminski , S. E. Munteanu , G. V. Zammit , and H. B. Menz , “Clinical Measures of Foot Posture and Ankle Joint Dorsiflexion Do Not Differ in Adults With and Without Plantar Heel Pain,” Scientific Reports 11, no. 1 (2021): 6451, 10.1038/s41598-021-85520-y. 33742026 PMC7979904

[35] E. von Elm , D. G. Altman , M. Egger , et al., “The Strengthening the Reporting of Observational Studies in Epidemiology (STROBE) Statement: Guidelines for Reporting Observational Studies,” Journal of Clinical Epidemiology 61, no. 4 (2008): 344–349, 10.1016/j.jclinepi.2007.11.008. 18313558

[36] D. Goutallier , J. M. Postel , J. Bernageau , L. Lavau , and M. C. Voisin , “Fatty Muscle Degeneration in Cuff Ruptures. Pre‐ and Postoperative Evaluation by CT Scan,” Clinical Orthopaedics and Related Research 304 (1994): 78–83, 10.1097/00003086-199407000-00014. 8020238

[37] N. D. Camarillo , R. Jimenez‐Silva , and F. T. Sheehan , “Using Bilateral Data in Controls and Patients With Bilateral and Unilateral Pathology Requires Increased Scrutiny,” Journal of Biomechanics 162 (2024): 111855, 10.1016/j.jbiomech.2023.111855. 37984294 PMC10843647

[38] H. B. Menz , “Analysis of Paired Data in Physical Therapy Research: Time to Stop Double‐Dipping?,” Journal of Orthopaedic & Sports Physical Therapy 35, no. 8 (2005): 477–478, 10.2519/jospt.2005.0108. 16187507

[39] J. F. Sallis , W. L. Haskell , P. D. Wood , et al., “Physical Activity Assessment Methodology in the Five‐City Project,” American Journal of Epidemiology 121, no. 1 (1985): 91–106, 10.1093/oxfordjournals.aje.a113987. 3964995

[40] M. T. Richardson , B. E. Ainsworth , D. R. Jacobs , and A. S. Leon , “Validation of the Stanford 7‐Day Recall to Assess Habitual Physical Activity,” Annals of Epidemiology 11, no. 2 (2001): 145–153, 10.1016/s1047-2797(00)00190-3. 11164131

[41] J. D. Stanczak , V. A. McLean , and L. Yao , “Atrophy of the Abductor Digiti Minimi Muscle: Marker of Neuropathic Heel Pain Syndrome?,” Radiology 221P (2001): 522.

[42] P. A. Butterworth , K. B. Landorf , S. E. Smith , and H. B. Menz , “The Association Between Body Mass Index and Musculoskeletal Foot Disorders: A Systematic Review,” Obesity Reviews 13, no. 7 (2012): 630–642, 10.1111/j.1467-789X.2012.00996.x. 22498495

[43] K. D. van Leeuwen , J. Rogers , T. Winzenberg , and M. van Middelkoop , “Higher Body Mass Index Is Associated With Plantar Fasciopathy/‘Plantar Fasciitis’: Systematic Review and Meta‐Analysis of Various Clinical and Imaging Risk Factors,” British Journal of Sports Medicine 50, no. 16 (2016): 972–981, 10.1136/bjsports-2015-094695. 26644427

[44] I. Khodarahmi and J. Fritz , “The Value of 3 Tesla Field Strength for Musculoskeletal Magnetic Resonance Imaging,” Investigative Radiology 56, no. 11 (2021): 749–763, 10.1097/RLI.0000000000000801. 34190717

