# Supplementary material for: Plantar Heel Pain Is Not Associated With Fatty Infiltration of the Abductor Digiti Minimi Muscle on Magnetic Resonance Imaging: A Cross‐Sectional Observational Study
Source: J Foot Ankle Res. 2026 Apr 25;19(2):e70155. doi: 10.1002/jfa2.70155 (PMC13110057; doi:10.1002/jfa2.70155)

**Supporting Information 1 – Data collection form for over-arching study**

**Note:** This form was used to collect data on participants in the initial over-arching study, including participant characteristic data.

**
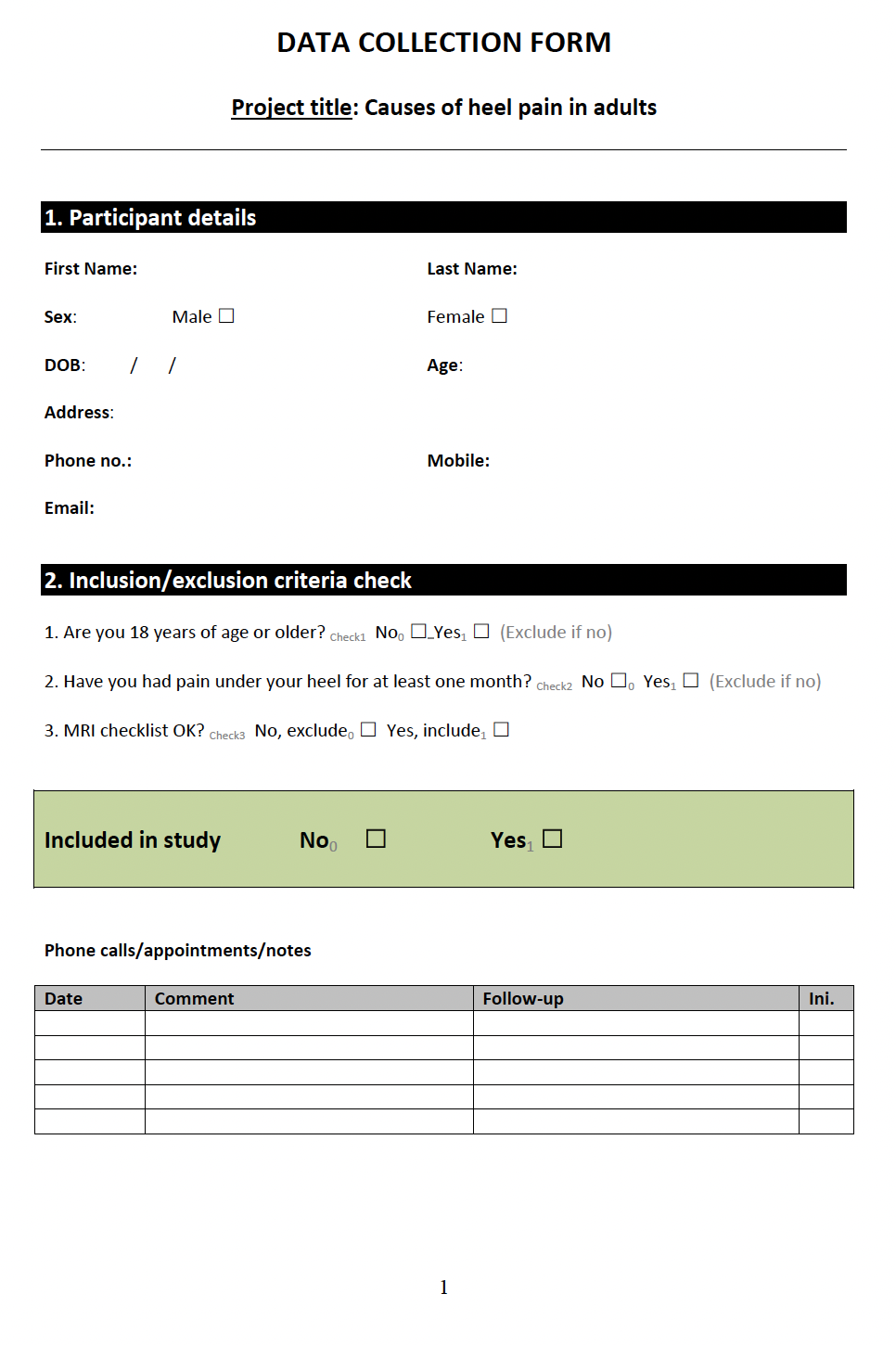
**


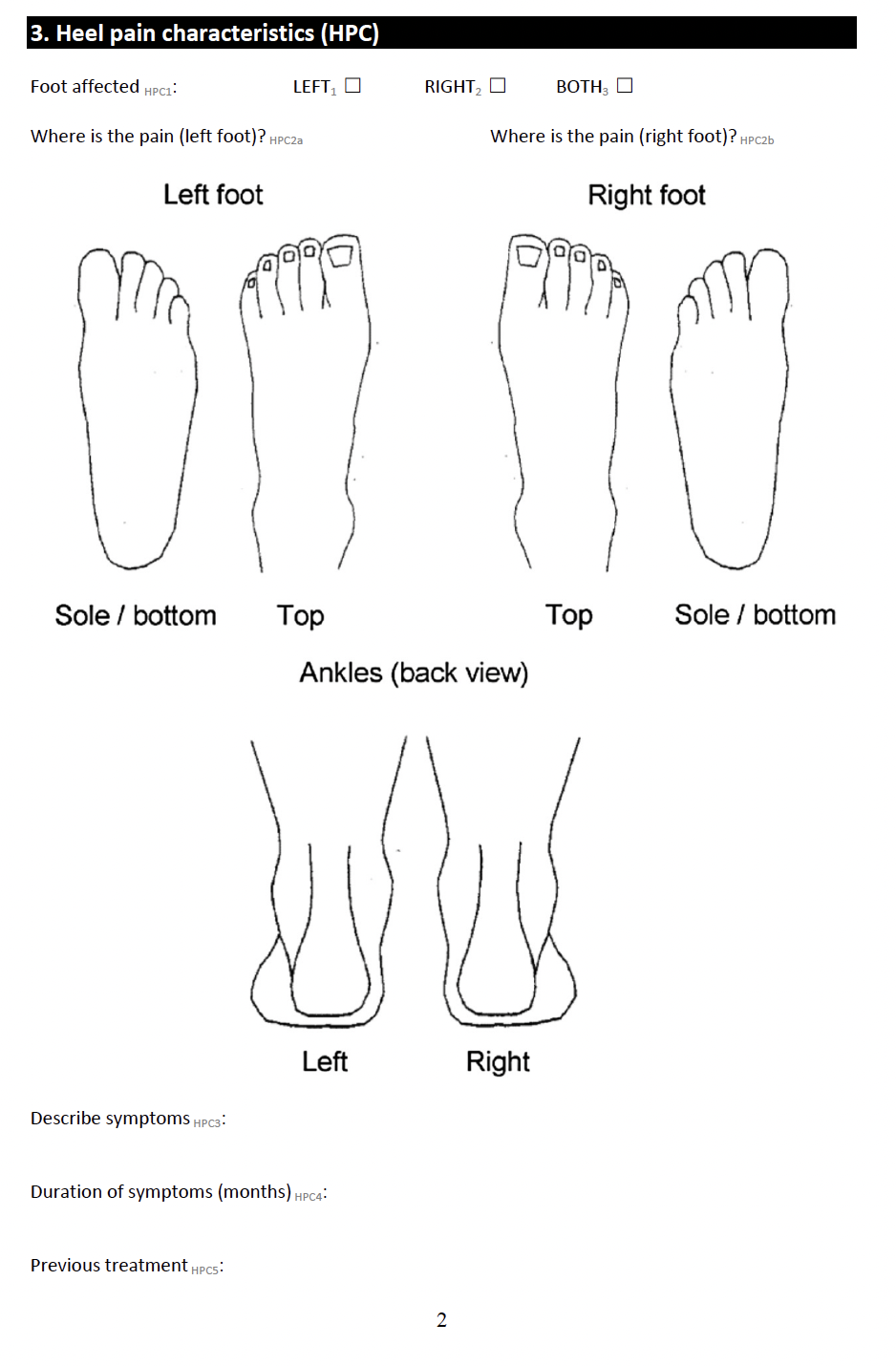


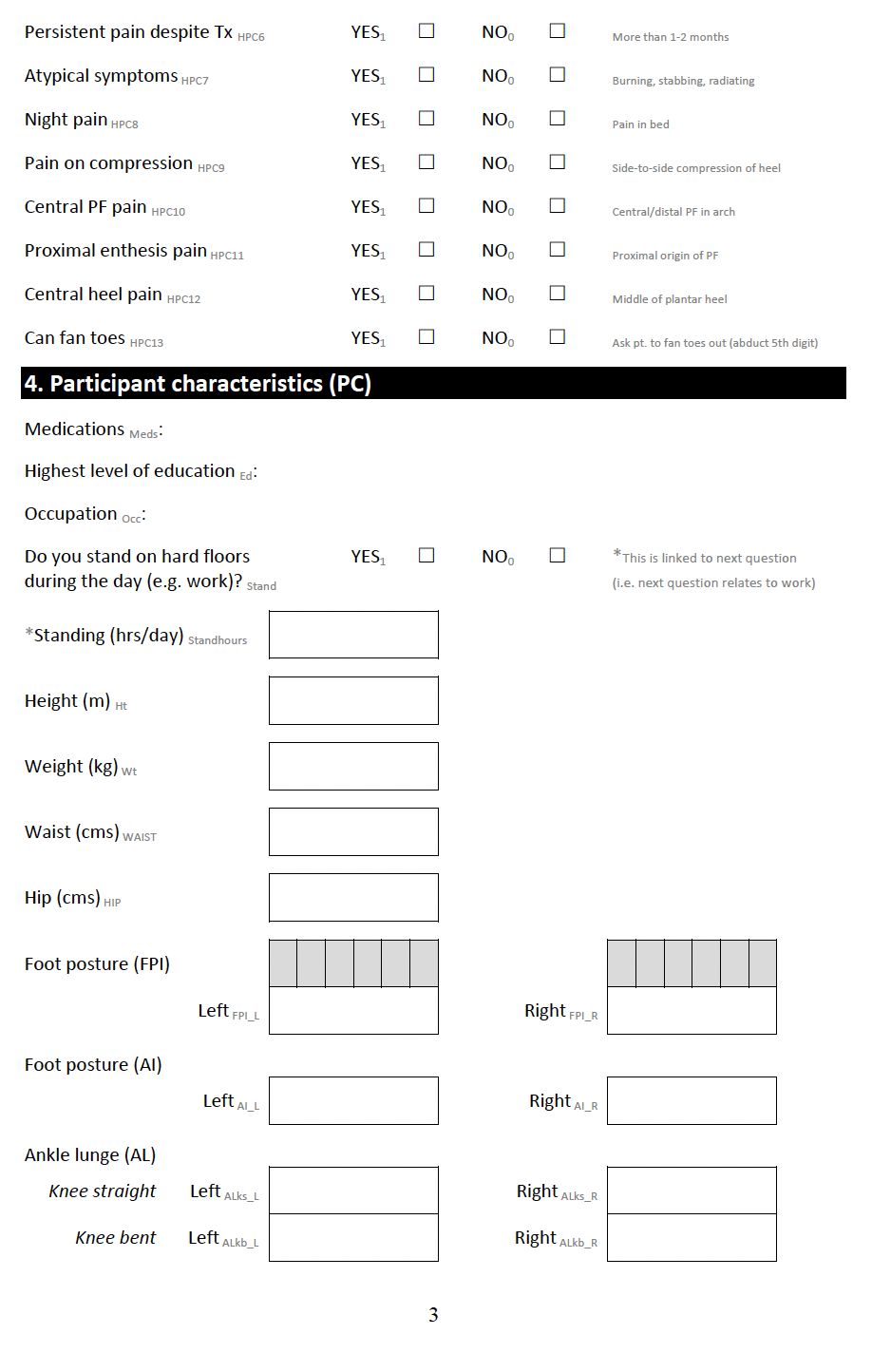


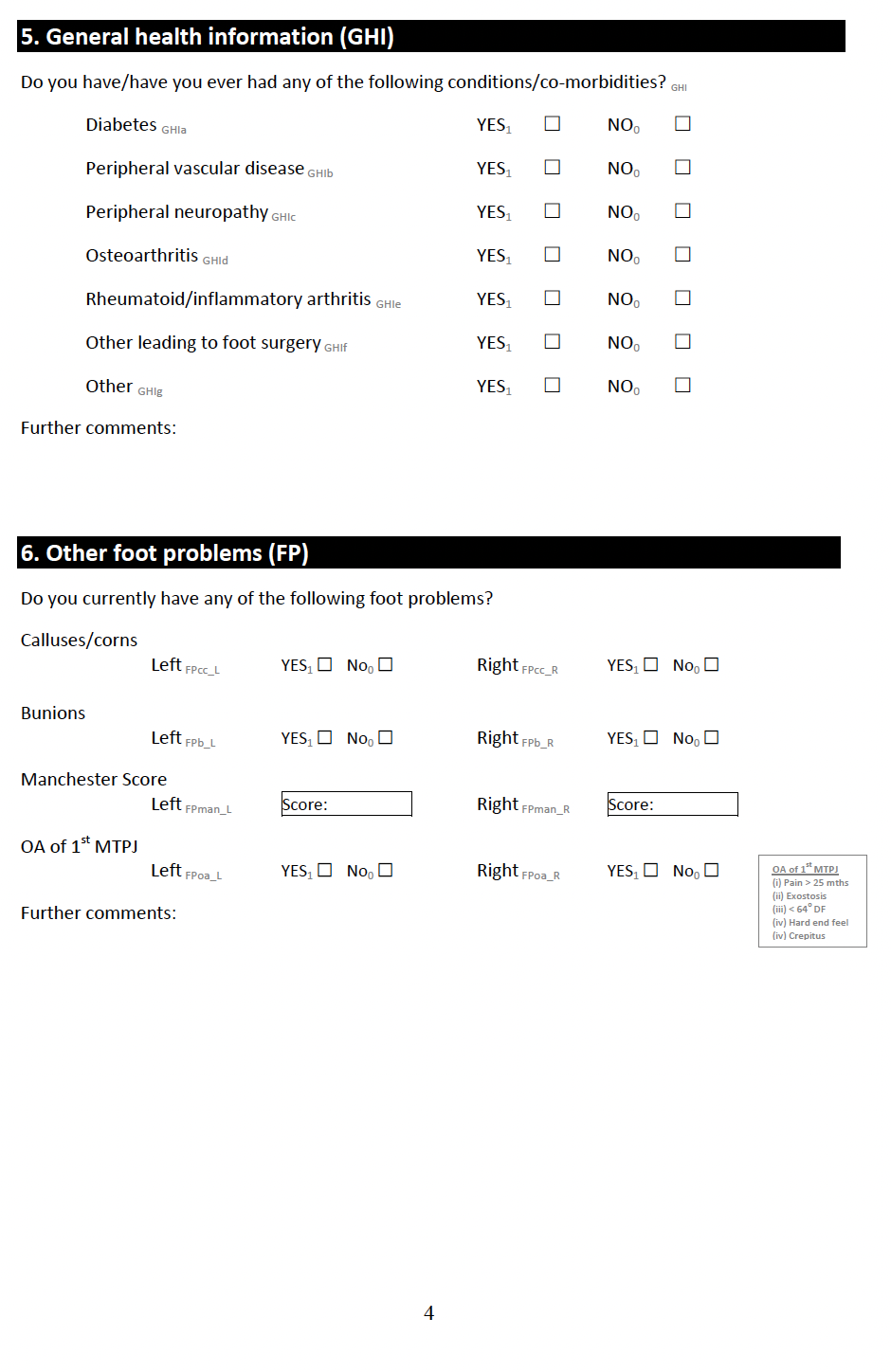


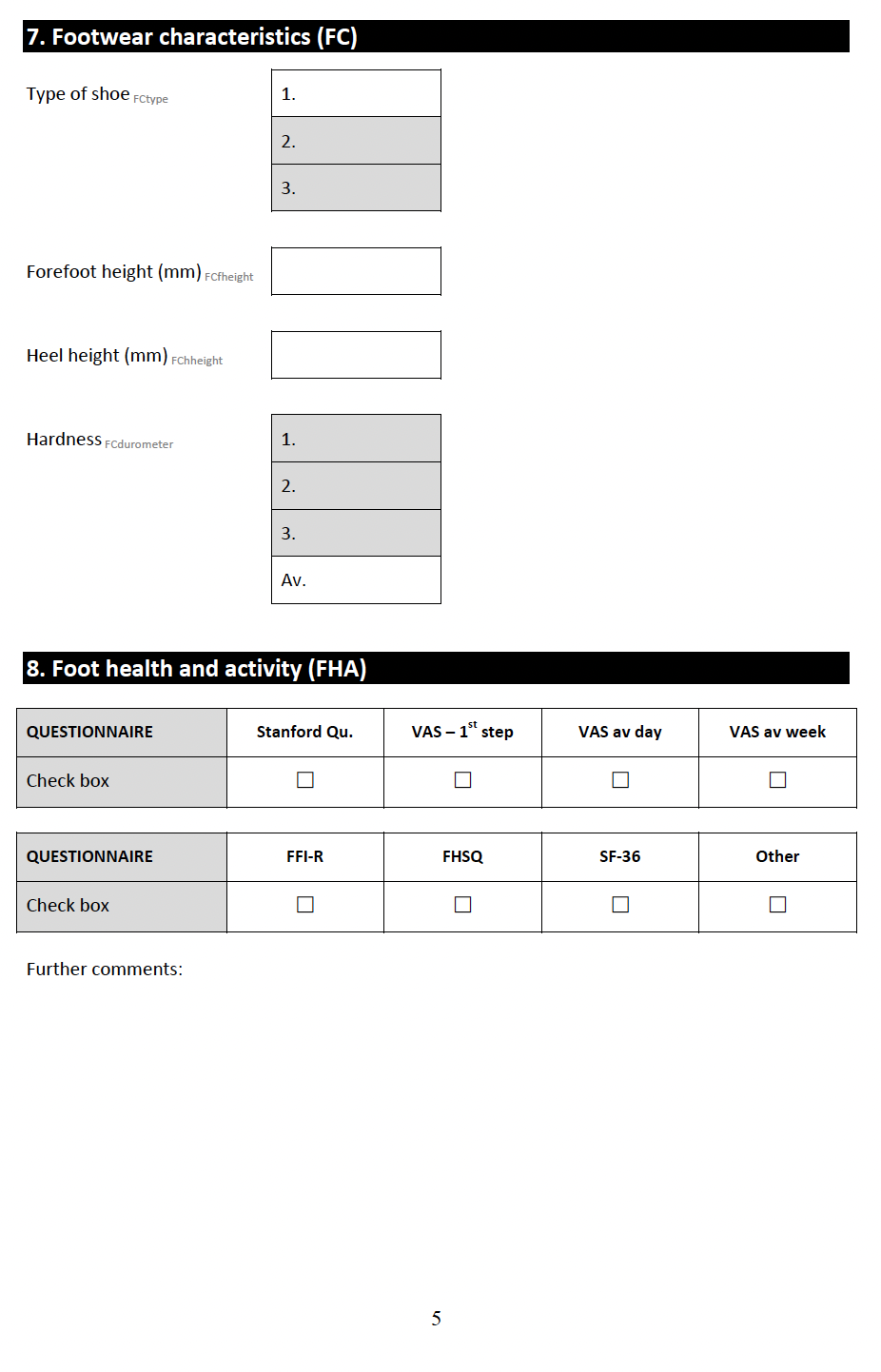

Supplement: Supplementary file 1 — Supporting Information S1 [file JFA2-19-e70155-s002.docx]
