# Supplementary material for: Plantar Heel Pain Is Not Associated With Fatty Infiltration of the Abductor Digiti Minimi Muscle on Magnetic Resonance Imaging: A Cross‐Sectional Observational Study
Source: J Foot Ankle Res. 2026 Apr 25;19(2):e70155. doi: 10.1002/jfa2.70155 (PMC13110057; doi:10.1002/jfa2.70155)
Supplement: Supplementary file 2 — Supporting Information S2 [file JFA2-19-e70155-s001.docx]

**Supporting Information 2 – Fatty infiltration grading tool**

**
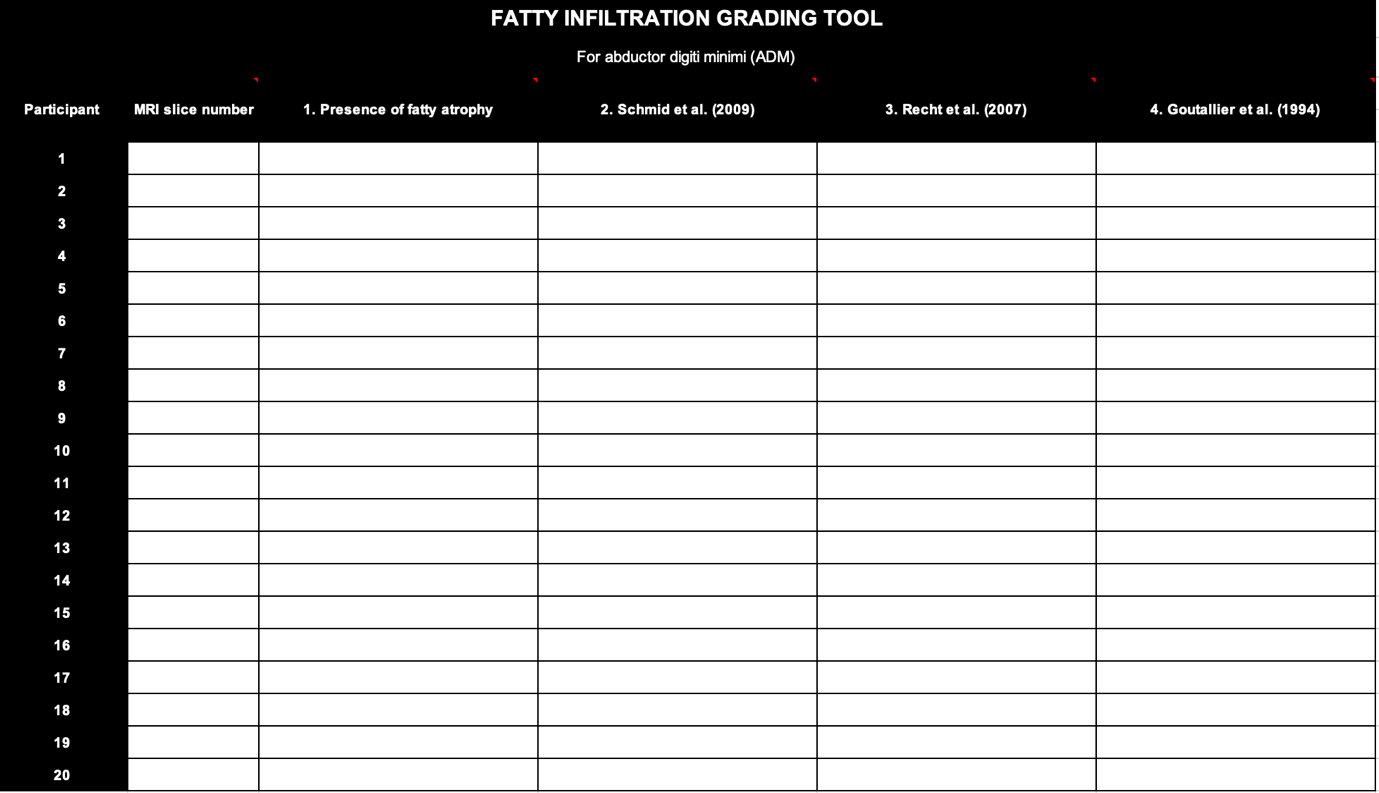
**

This tool was custom developed on a Microsoft Excel spreadsheet. Each participant was assessed according to the four grading scales that were initially considered.

**
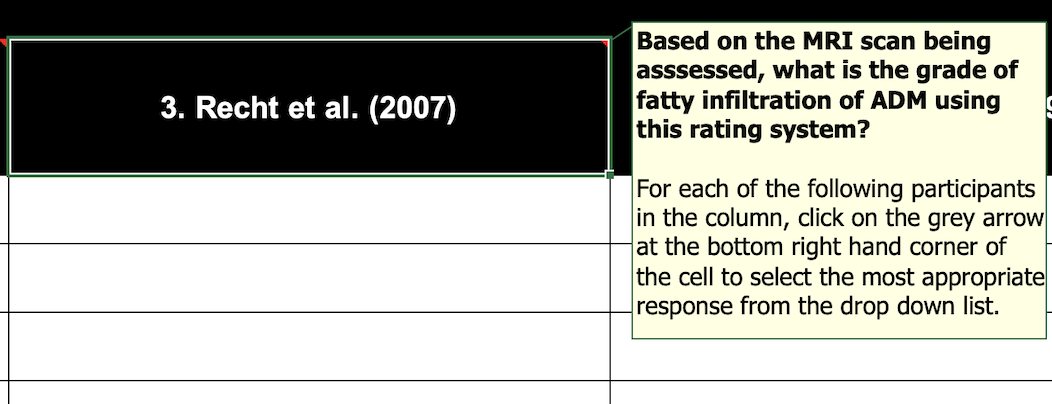
**

The title cell of each grading scale column contained an instruction note for the assessor to follow (see above example provided using the Recht et al. grading scale)**.**

**
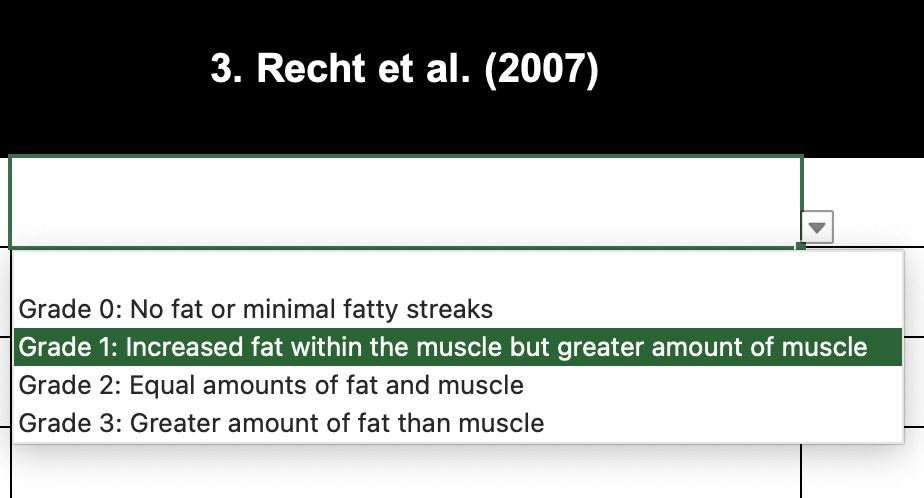
**

Each cell that corresponded to each participant row had a forced choice response that the assessor needed to choose from, according to the specific grading scale in that column (see above example using Recht et al. grading scale).
